# Bridging Gaps in Long COVID Therapy: A Review

**DOI:** 10.2174/0109298673389691250709181809

**Published:** 2025-07-30

**Authors:** Wassan Nori, Zina Abdullah Hussein, Roaa Mokram Hamed, Mufeed Taha, Alexandru Cosmin Pantazi

**Affiliations:** 1 Department of Obstetrics and Gynecology, Al-Mustansiriya University, Baghdad, Iraq;; 2 The National Center of Hematology, Al-Mustansiriya University, Baghdad, Iraq;; 3 Department of Medicine, University of Kirkuk, Kirkuk, Iraq;; 4 Department of Pediatrics, Ovidius University, Constanta, Romania

**Keywords:** Long COVID-19, SARS-CoV-2, non-pharmacological options, pharmacological options, biomarkers, Google scholar

## Abstract

**Introduction:**

Long COVID-19 (LC) is a condition that follows SARS-CoV-2, an acute infection defined by persistent fatigue, dyspnea, and impaired cognitive function. LC presents a complex array that imposes ongoing challenges on global health, patients' quality of life, and functional capacity. Many inconsistencies surround its pathophysiology, diagnosis, prevention, and treatment. This review aims to cover missed gaps in LC with a special focus on therapeutic strategies concerning non-pharmacological, pharmacological, experimental, and innovative approaches for better patient management and outcomes, as well as to evaluate their effectiveness and guide future research.

**Methods:**

An online search was conducted using five digital repositories: PubMed, Scopus, Google Scholar, Web of Science, and the Cochrane Library. A combination of keywords associated with LC therapy was employed: “long COVID, “pharmacological options,” “non-pharmacological options,” “innovative strategies,” “experimental”, and” quality of life (QOL).” Relevant data were extracted and synthesized to categorize therapeutic approaches into subtypes. A critical analysis was conducted on their mechanism of action, indication, outcome, and limitations.

**Results:**

The pooled prevalence of LC was 42%, and the symptom duration ranged from 3 months to 2 years. The most important risk factors for LC were female sex, unvaccinated status, and cases with co-morbidities. Diagnosis of LC was challenging due to a lack of diagnostic standardization and reliable biomarkers.

**Discussion:**

Non-pharmacological strategies were employed first, showing diverse efficacies; however, the reported literature was hindered by small sampling. Pharmacological agents show promising results but need further validation. Experimental and innovative strategies need longer studies and validations.

**Conclusion:**

LC has imposed a significant burden on community health, necessitating the appropriate allocation of health resources and community support. Preventive and therapeutic interventions show promise, but the variability in patient response underscores the need for personalized approaches and more well-designed trials. Collaborative research and multi-disciplinary teams are needed to mitigate the long-term effects of LC and improve patient outcomes.

## 1 INTRODUCTION

The COVID-19 pandemic has imposed profound and far-reaching implications on global health, with unprecedented mortality, morbidity, and societal isolation since the declaration of the COVID pandemic in 2019 [1]. Vaccination campaigns and public measures such as social distancing have played pivotal roles in mitigating the pandemic's acute impact [2]. However, the long-term consequences of COVID continue to pose significant challenges, emphasizing the need for sustained scientific inquiry and innovation [3].

While the majority of COVID-19 cases (up to 60%) recover from acute symptoms within one month, a substantial proportion (up to 40%) suffer from persistent and diverse symptoms that collectively constitute post-acute sequelae of SARS-CoV-2 infection or Long COVID (LC), imposing significant health challenges [4, 5].

LC is a complex and heterogeneous condition that encompasses a wide range of symptoms, including fatigue, dyspnea, chest pain, myalgia, loss of concentration, depression, and insomnia. These symptoms have been reported in diverse presentations impacting different populations, and they inversely affect the quality of life (QOL) and functional capacity of the affected individual [6].

The (WHO) definition for LC is the appearance of unexplained symptoms that last at least two months after three months following the primary SARS-CoV-2 infection [7]. The estimated pooled prevalence of LC is 42%, with symptom duration reported to range from 6 months post the acute infection, based on some reports and extending to 1-2 years according to others [8, 9]. Notably, many of the recovered COVID-19 cases reported persistent symptoms. Fatigue, cough, and cognitive decline were the most commonly reported symptoms [10]. Emerging evidence highlights significant disparities in the risk of developing LC, with racial, gender, and socioeconomic differences playing critical roles. In addition, preexisting co-morbidities (such as hypertension and diabetes), advanced age, and high body mass index (BMI) further escalate the vulnerability to LC [11-13]. Despite extensive work, there are still missing gaps in the LC pathophysiology, and inconsistencies in the reported symptoms and investigation. These were further confounded by a lack of standardized diagnostic studies and validated biomarkers, which makes LC confirmation a diagnosis of exclusion, complicating early detection and management.

Currently, managing LC includes the use of pharmacological agents (such as anti-inflammatory and antiviral) and non-pharmacological agents (such as lifestyle modifications and rehabilitation) [1, 4]. These methods face several limitations: the lack of standardized protocols for treatment owing to symptom diversity and the lack of specific biomarkers that hinder universal diagnostic tests. In addition, limited evidence supports the efficacy of available therapy, which is further hampered by accessibility issues and the illness's social impact, hampering effective care [3, 10].

The burden of long COVID on the health system and economy is extensive. Affected individuals experienced increased utilization of healthcare services, including rehabilitation programs and mental health support, suffered reduced work productivity, and long-term disability. A significant financial burden was raised by LC, including higher absenteeism rates, earlier retirement, and higher demand for prolonged medical care and interventions to address increased complications [1, 4, 10].

The current therapeutic options for LC remain limited, especially for fatigue management, which underscores the need to develop effective therapeutic strategies for affected cases.

This review aims to:

- Examine the current evidence on LC pathophysiology, predisposing factors, investigation paradigms, and diagnostic challenges. By enhancing the current understanding of LC, we aim to guide future research direction and enhance evidence-based clinical decisions.
- Critically discussed available therapeutic strategies focused on:
  1. Define major subtypes of therapeutic intervention for LC cases, describing supporting evidence and each method's disadvantages and side effects.
  2. Explore innovative and experimental methods to guide future research.
  3. To discuss the LC prognosis, the burden on global health, and future recommendations.

By bringing a comprehensive critique of patient therapeutic strategies, we aim to bring up-to-date knowledge to improve patient care and outcomes and to identify key research gaps to guide future works and support evidence-based decisions.

## 2 METHODS

This comprehensive review examines the evidence on various aspects of LC, including its pathophysiology, risk factors, presenting symptoms, diagnostic challenges, complications, and management strategies. The focus was primarily on recent therapeutic strategies for LC, including non-pharmacological (NP), pharmacological, and innovative therapies. A thorough search of peer-reviewed publications was conducted across five digital repositories: PubMed, Scopus, Google Scholar, Web of Science, and the Cochrane Library. The search included publications up to 1/1/2025, ensuring the inclusion of the most recent studies. A combination of keywords and subject headings associated with LC treatment was employed: “long COVID “, “Pharmacological options”, “non-pharmacological options”,” innovative strategies”, and” quality of life (QOL)”. Keywords were combined by Boolean operators like “AND” and “OR” to improve search precision and breadth. We included articles that matched our keywords and were published in English in indexed journals within our set time frame.

Exclusion was made to any studies that were written in a non-English language, case reports, and articles that did not address the parameters of interest. For each included article, we gathered data about the authors’ names, publication year, intervention type (non-pharmacological, pharmacological, or innovative), the suggested mechanism of action, and perceived benefit. The extracted data were synthesized to create therapeutic approaches and categorized according to subtypes, and their outcome and limitations were analyzed. We have summarized the study methodology, inclusion and exclusion criteria, and the overall structure of the reviews in Fig. (**1**).

## 3 LITERATURE REVIEW RESULTS

### 3.1 Long Covid Pathophysiology

**Fig. (2):**
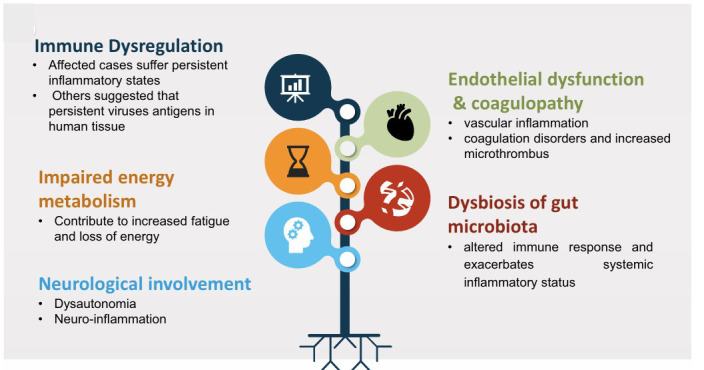
The suggestion mechanism that underlies long COVID.

The pathophysiology of Long COVID, also known as post-acute sequelae of SARS-CoV-2 infection (PASC), is complex and multifaceted. Current research has identified several potential mechanisms for its development; (Fig. **2**).

#### 3.1.1 Immune Dysregulation and Viral Persistence

- Affected cases suffer from persistent inflammatory status and altered cytokine profiles. Disturbed immunological response leads to autoimmunity reactions and ongoing symptoms [14].
- Others suggested that persistent virus antigens in human tissue will trigger continuous antigenic stimuli that cause ongoing immune activation and inflammation [15-17]. Here, anti-inflammatory and immune-modulating agents added to antiviral drugs were employed as therapeutic strategies to address this point.

#### 3.1.2 Endothelial Dysfunction and Coagulopathy

- COVID-19 can trigger vascular inflammation by directly infecting endothelial cells, reducing blood supply, and impairing flow. The latter will contribute to fatigue and impaired cognition [18].
- The invading viruses were linked to many coagulation disorders and increased microthrombus formation, impeding oxygen delivery and exacerbating symptoms [19]. For that, anticoagulants and endothelial vascular agents are beneficial therapy.

#### 3.1.3 Neurological Involvement

- Dysautonomia: Autonomic central nervous system (CNS) dysfunction. Some cases have reported irregularity of the autonomic CNS manifested by orthostatic intolerance, irregular heart rhythm, and GIT disturbances [20].
- Neuro-inflammation: Persistent inflammation within the CNS among cases with LC may be reflected by cognitive deficits, known as brain fog [20, 21]. Anti-inflammatory drugs, rehabilitation, and neuromodulation were used to improve therapeutic outcomes.

#### 3.1.4 Impaired Energy Metabolism

- Mitochondrial dysfunction and impaired energy production were suggested mechanisms for increased fatigue and energy loss among cases with LC [22]. Nutritional interventions, metabolic support, and mitochondrial targeting agents were employed to enhance recovery.

#### 3.1.5 Dysbiosis of Gut Microbiota

- Gut dysbiosis (an altered healthy balance of gut micro-organisms, such as bacteria, viruses, and fungi) contributes to an altered immune response and exacerbates systemic inflammatory status among affected cases [23]. The use of probiotics and microbiome-targeted therapy can reduce immunological dysregulation and reduce the inflammation status.

### 3.2 Risk Factors For Long COVID

**Fig. (3):**
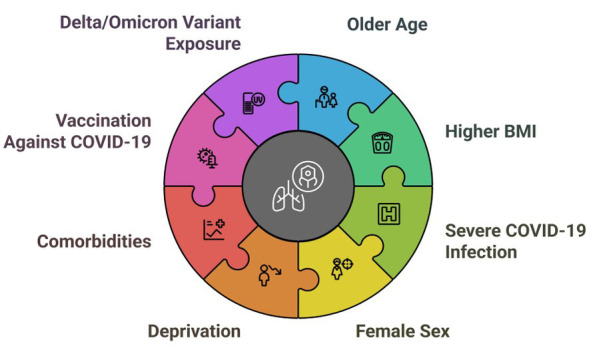
Risk factors for developing long COVID.

An interplay of patients' demographics, social criteria, and co-morbidities escalates the risk of long-term COVID; (Fig. **3**). Current research suggests that vaccination and a male gender offer protection; on the other hand, advanced age, severe primary COVID-19 infection, and female gender increase the odds of long-term COVID-19 [24].

- ***Gender***: Females are more prone to long COVID by 1.5 -2 times. Some discussed an odds ratio (OR) of 1.8 based on patient gender [25]. Reasons include hormonal factors such as estrogen, which tend to boost immune responses and exaggerate inflammation during recovery. Having two XX chromosomes will increase immune-linked genetic activity, which contributes to chronic inflammation and prolonged symptoms. Females have increased susceptibility to immune diseases and are more likely to report them and seek help [26].
- ***Age*:** Younger ages show less likelihood of getting long-term COVID-19, while older ages show heightened risk, particularly those above 65 years [25, 27]. Some studies suggest that middle-aged people have an OR of 1.03 per year to develop LC.
- ***Body mass index (BMI):*** Obese individuals are more likely to have LC. Obesity interferes with and intensifies inflammatory responses. Additionally, it is linked to metabolic dysregulation and, most importantly, respiratory problems. Taken together, obese individuals suffer higher odds for LC, OR ranging from 1.15-2.15 [28, 29].
- ***Patients' co-morbidities***: Having three morbidities will increase the risk, with an OR of 3.5. The most common related co-comorbidities include hypertension, diabetes, chronic lung disease, depression, and obesity [30-32].
- ***Vaccination status***: Vaccination reduces the odds of having LC (OR:0.59). The number of vaccination doses inversely correlates with the risk of LC, as it mitigates the inflammatory response and lowers persistent infections. This was especially noticed during the Omicron and delta strains [33-36].
- ***The severity of primary COVID Infection*:** The severity of acute infection was positively correlated with the risk of LC, with an estimated increased risk (OR 2.4); it can be attributed to exaggerated inflammatory responses, higher injury, and viral load. Additionally, the associated coagulation changes will amplify LC risk [37, 38].
- ***Blood types***: Having a blood group of O, AB, and Rh-negative blood types is protective against LC; however, the exact mechanism is unclear. It is worth mentioning that O blood type was linked with a lower risk of COVID-19 infection and adverse outcomes (OR 0.88 and 0.82), respectively [11, 39].
- ***Socioeconomic factors:*** Lower socioeconomic status was a risk factor for LC cardiovascular complications, with an overall hazard risk of 1.2; the risk was higher for hospitalized cases compared to non-hospitalized patients (1.9 *vs* 0.8). No association was reported for respiratory and general LC symptoms [40]. The interplay of gender, vaccination status, patient co-morbidity, and the diversity in the incident across diverse populations suggests that LC is not a uniform condition but a multi-phenotype-diverse syndrome. Assessing and understanding LC through intersectionality is crucial as it allows more precise, equitable intervention and targeted treatment approaches, especially for high-risk groups. Accordingly, large cohort studies are needed to ensure a tailored therapeutic strategy rather than a one-size-fits-all model.

## 3.3 Symptoms of Long COVID

The symptoms of LC are diverse and described in Table **1**, with the reported incidence and description [41-48]. It is worth saying that many symptoms (fatigue, cognitive impairment, impaired subjective well-being) may persist in some recovered cases for up to 2 years, impairing the quality of life (QOL) [49]. Another study reported gradual improvement in the symptoms, which was affected by the severity of the primary infection and preexisting health conditions [50].

### 3.4 Diagnosis of Long COVID and Follow-Up

The condition is characterized by multi-organ involvement with diverse manifestations, including (respiratory, neurological, gastrointestinal (GIT), cardiovascular, …), leading to heterogenicity in symptoms [41-45]. This is further confounded by the lack of universal diagnostic symptoms, making diagnosis challenging. The medical history of the case may be suggestive; however, not all patients had a confirmed history of COVID-19 infection [4, 5]. A clinical examination must confirm any associated complications or exclude other differential diagnoses. Likewise, tests needed to exclude other diagnoses and confirm long COVID may vary according to the patient's complaint [6]. These tests may include blood tests, imaging tests, and electrocardiography. It is worth noting that some of these tests will be normal even in the presence of long COVID.

#### 3.4.1 Blood Test

Many studies have pointed out that inflammatory markers tend to rise in LC, including interleukin-6, C-reactive protein, and tumor necrotic factor-alpha; these may serve as diagnostic biomarkers [51, 52]. Other studies discussed distinct immune profiling among affected cases, including increased cytotoxic immune cells and altered antibody responses (higher SARS- CoV-2 and EBV antibodies were noticed [53].

Furthermore, affected cases showed marked differences in some hormones, mainly cortisol. It was shown that systemic levels of cortisol are reduced in LC cases compared to controls. Klein, Jon, *et al.*'s study discussed that by integrating immunological profiling and demographics of COVID-19 patients, a reliable prediction of LC was made by machine learning. Thus improving the care for those with a higher risk [54].

There were a number of biomarkers that could predict the degree of lung injury and occurrence of LC, including High Lactate Dehydrogenase (LDH), reduced level of T-cells, and elevated interleukin-6. Additionally, elevated D-dimer at admission was an independent predictor for lung injury within 3 months [55].

#### 3.4.2 Imaging Studies

A CT scan is the investigation of choice for evaluating lung injuries and follow-up. There are several cases where radiological signs were shown even when the cases were asymptomatic. Ground glass opacities and fibrotic-like changes are the most frequently reported [56].

Most of these changes tend to resolve in 3-6 months following the acute infection. Still, some tend to be persistent and should be evaluated carefully. Persistent changes are more likely to be seen in cases of severe infection, mechanical ventilation, and extensive lung injuries. Extended follow-up is recommended for those cases, particularly if they show persistent or newer symptoms. Computerized CT scan (CT) is superior to chest X-ray (CXR) and ultrasound (US) in follow-up [57].

#### 3.4.3 Test for Lung Injury if any Reported

In patients reporting respiratory symptoms, a history of severe infection, or admission to the intensive care unit (ICU), the recommended test is the pulmonary function test (PFT), which should be within 3 months following recovery [58]. If the symptoms persist or worsen, a re-test is recommended every 3-6 months. A multi-disciplinary team, including pulmonologists and rehabilitation specialists, is recommended for management. For follow-up, recommended tests include DLCO (Diffusing capacity of the lung for Carbon Monoxide) and advanced imaging [59].

#### 3.4.4 Test for cardiovascular disease in Long COVID

These include biochemical, physiological, and imaging tests. Biochemical tests include NT-pro BNP levels (N-terminal pro-B-type natriuretic peptide), a common biomarker for assessing cardiac function during stress and heart failure. Physiological tests include ECG and cardiopulmonary exercise testing (CPET). The latter examines heart anaerobic capacity, although its results may not correlate with the patient's symptoms [60]. Imaging tests include Echo, CXR, CT, and cardiac MRI, with its special subtype, Cardiovascular magnetic resonance imaging (CMR) [43].

#### 3.4.5 Test for Psychological Issues and Quality of Life (QOL)

Some of the most commonly used tools are HADS (Hospital Anxiety and Depression Scale), SF-36 (Short Form-36), and SGRQ (George's Respiratory Questionnaire) [61].

However, some suggested that patient interviews are more practical in evaluation. There is a need for standardized, rationalized approaches to assess LC burdens and patient well-being. Follow-up in the first month after hospitalization is critical to capture acute psychiatric symptoms. Extended follow-up and assessment of QOL are needed to mitigate persistent issues [62]. It is worth noting that support from the family and healthcare giver, and engagement in rehabilitation and support groups *via* telemedicine are all good options for patient support [61].

### 3.5 Prevention of Long COVID

Complete prevention of LC remains challenging. Currently, there is no guaranteed intervention offering full protection. However, the most effective strategy to reduce long-term COVID is by preventing the initial infection; having a vaccine and the patient's own immunity may not completely protect against the infection, but may limit the acute and chronic viral sequelae [63-65]. The primary infection severity is a risk for LC and can be mitigated by using an antiviral drug that will help viral clearance and reduce viral shedding once it is reactivated [66].

Vaccination is another important way to avoid severe COVID-19 infection. Vaccines enhance immunity, reduce viral persistence, and reduce LC odds. The reported incidence of LC among vaccinated cases was 3.5%, underscoring its critical role [67]. Vaccinated cases had protection against LC with an OR of (0.54;95% CI =0.29-0.98) *versus* unvaccinated cases [33].

A healthy lifestyle, balanced nutrition, regular sports, and enough sleep reduce the risk by half by enhancing immunity and reducing inflammation [46]. Preventive strategies such as masking, social distancing, and washing hands are complementary yet important to adhere to. More research is needed to optimize prevention and address challenges imposed by LC [68].

### 3.6 Treatment of Long COVID

Long COVID needs a multi-disciplinary approach encompassing medical management, mental support, and community initiatives to mitigate its long-term effects. There are non-pharmacological and pharmacological approaches to treatment [69]. Many subtypes of non-pharmacological approaches that were examined in practice with variable efficacy [70-88] are summarized in Table **2**.

While exercise-based interventions and acupuncture seem to have promising efficacy, other, like complementary and alternative medicine, seems to lack strong supporting evidence.

Non-pharmacological strategies alleviate LC symptoms through multiple mechanisms. On the molecular level, exercise reduces systemic inflammation, enhances mitochondrial function, and supports symptom mitigation. Additionally, it promotes cardiovascular reconditioning, increases cerebral blood flow, enhances neuroplasticity, and improves cognitive and physical recovery [72-74]. Acupuncture exerts inflammatory and cytokine regulatory effects, relieving pain and enhancing endorphin release. Further, acupuncture modulates neuroimmune pathways and improves microcirculation, thus alleviating pain and fatigue, two common complaints in LC cases [82].

Significant gaps in current knowledge urge well-designed, rigorous trials. Furthermore, a holistic approach that integrates evidence-based practice with strategies to enhance symptomatic relief, such as promoting a healthy lifestyle, adequate sleep, and a balanced diet, is essential to optimize patient care [70].

As for pharmacological therapeutic strategies [89-98], they were summarized in Table **3**. Finally, experimental and innovative strategies [99-109] are described in Table **4**.

### 3.7 Complications of Long COVID

**Fig. (4):**
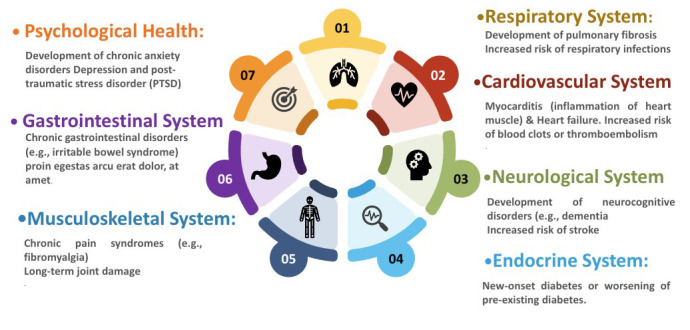
Full spectrum of long COVID complications.

Long COVID has imposed unprecedented challenges on affected cases and their communities. LC had a long list of complications that span many organs, including respiratory, cardiovascular, neurological, and mental health (Fig. **4**) [110].

Many unspecified symptoms tend to persist among LC cases, which inversely impacts QOL, which was significantly reduced among LC cases with a hazard ratio of 0.94 and an odds ratio for mortality of 4.7 [110, 111]. Moreover, LC had profoundly reduced social and occupational productivity, which led to prolonged absence from work; this was confounded by the psychological implications, which further confounded the impact of LC on humankind [75]. There is no doubt that long-term COVID-19 imposes significant challenges on community health. Moreover, it impacted the social and work output of affected individuals, not to mention their mental health [112].

### 3.8 Prognosis of Long COVID

Many factors affect LC prognosis [113]; poor prognostic indicators include:

1. Female gender women tend to suffer from LC more than men, with an OR of 1.52; 95% CI 1.27–1.82) based on Maglietta *et al.*'s meta-analysis [36, 114].
2. The severity of the primary infection: cases with severe primary infection tend to have worse outcomes with an OR of (1.66, 95% CI 1.03–2.68) [114, 115].
3. Concurrent co-morbidities: Patients with asthma and diabetes tend to have challenging recovery trajectories [113].
4. New-onset neurological symptoms, particularly fatigue and cognitive impairment, are among the worst in prognosis and may take years or even a lifetime to resolve with an OR of (1.67, 95% CI 1.21–2.29) [114, 116]. The time needed for recovery from LC symptoms varies; still, many agree on an approximate time frame for clearing them, as clarified in Table **5** [117-122].

Management of CNS complications in LC is challenging, as it needs a tailored patient approach, a multitherapy rather than monotherapy, and it should address patients' subtypes and underlying mechanisms [123-125]. The available therapeutic approach aims to alleviate patient symptoms and enhance QOL, including non-pharmacological and pharmacological strategies.

The non-pharmacological approach is crucial in addressing neurological LC symptoms that persist long after the infection. Of these interventions, we have cognitive rehabilitation, such as goal management training, which showed optimistic results in enhancing executive functions and attention deficit linked with LC [126-129].

Psychological support is also used in managing some of LC's symptoms, such as depression, anxiety, and post-traumatic stress symptoms. Integrated psychological support, structural mental health intervention, and counselling significantly contribute to patients' recovery and better QOL [130].

As for the pharmacological interventions, these include drugs that are used for “symptomatic relief”, including low-dose propranolol, clonidine, SSRIs, and low-dose tricyclic antidepressants [38]. Other drugs that aim to improve cerebral circulation include fludrocortisone, midodrine, and pyridostigmine [38, 131]. Stimulants such as modafinil have shown optimistic improvement in cognitive dysfunction and fatigue [131]. While some innovative therapies, such as intravenous immunoglobulin, are being tested for post-autonomic and autoimmune complications [104, 105], they need further validation. Future research should evaluate treatment efficacy in the long term and explore newer biomarkers and diagnostic criteria for LC [132].

## 4 DISCUSSION

Identification of predictors of LC and patients at higher risk will enable laying out effective health strategies and resources to address their needs [133]. Vaccination was consistently identified as a protective factor against the development of LC. This point holds critical implications for public health strategy and policymakers [134].

Despite ongoing research, our knowledge is far from complete. The duration required for LC symptoms to resolve varies widely across studies. While some reported recovery periods of weeks, others suggested persistent symptoms for up to 2 years [38, 114, 135]. Another major limitation of LC research is the inconsistency and lack of standardization in defining and measuring clinical trial outcomes.

### 4.1 Suggested Areas for Future Research

Future studies should unveil the complexity and heterogeneity of LC risk factors and the persistence of its symptoms, particularly fatigue and cognitive decline.

Several emerging hypotheses were suggested to explain the diverse manifestations of long-term COVID-19; however, the full aspect of the LC pathogeny remains incompletely understood. To date, critical gaps persist, with many mechanisms still unresolved, and no single hypothesis can fully explain the LC complexity [136, 137].

One hypothesis has addressed the interplay between viral persistence, immune dysregulation, and CNS involvement. It is suggested that the reactivation of latent viral infections could trigger long COVID, such as the Epstein-Barr virus, which triggers a chronic inflammatory state [138].

Phillips & Williams's study discussed that LC is fundamentally a CNS disease involving a sustained neurological alteration affecting brain function. This hypothesis is supported by autonomic dysfunction and neuroinflammation [139].

Wong *et al.* suggested that deposited viral remnants in the patients' GIT cause serotonin deficiency; the latter is responsible for sleep and cognitive function. Its deficiency may explain many of the long-term COVID-19 symptoms [140].

Joffe *et al.* postulated that LC symptoms may be attributed to functional somatic symptom disorder. In this disorder, the psychological factors trigger physical symptoms and exacerbate their existence, making them hard to treat. This interaction of psychological and physical health makes managing this disorder challenging [141].

Ongoing research should prioritize enhancing our understanding of LC pathophysiology and complications to identify optimal, safer therapeutic strategies and develop effective preventive interventions to mitigate its long-term impact [142, 143]. Prediction models for LC development show promise but require ongoing validation and refinement to guide medical decision-making.

While pharmacological options in LC treatment are being used, there are long-term risks linked to their use that should be evaluated. For example, steroids carry the risk of osteoporosis and immune suppression in the long run (920. Antidepressants, on the other hand, can cause dependency and CVS side effects [97]. We must acknowledge that managing LC is hindered by symptom heterogeneity and variability in the clinical response, superadded by long-term use's side effects [144].

Having said that, we need a standardized guideline and precision medicine to mitigate the risks of polypharmacy. Future research should examine long-term drug safety and conduct comparative efficacy trials to reach robust evidence for the drugs used.

Another area of future research is the challenges faced by non-pharmacological treatments in LC. The first is their limited accessibility, which hinders their widespread implementation. Second, financial issues compromise patients' adherence to therapy, and finally, LC's diversity of symptoms makes it difficult for a single approach to be universally effective [72-75, 78].

The role of digital health in LC is worthy of assessment, whether in telehealth or online rehabilitation programs. Telemedicine (TM) had a critical role during and after the COVID-19 pandemic. TM programs have enhanced accessibility and continuity of care for LC cases, especially in underserved areas. TM has enabled remote monitoring and patient education with the advantage of multi-disciplinary care [145].

Moreover, virtual rehabilitation programs gave guided therapy for the physical and psychological symptoms of LC. Still, there are challenges facing TM implementation in practice; these include digital literacy, internet access challenges and lack of supporting technology [146]. Long-term evaluation is needed for their efficacy to determine how they can be integrated into everyday practice.

Finally, many of the therapeutic interventions and studies included in this review were hindered by small sample sizes and insufficient follow-up durations. Addressing these points will deepen our insight into LC pathophysiology and develop effective and safe therapeutic strategies. Since the LC era is an entirely new area of research, we recommend more long-term comparative studies to address the LC knowledge gap, which is constricted by the current state of available evidence.

### 4.2 Study Limitations

An exclusion of non-English articles may create study bias, added to the heterogenicity of included studies in the current analysis [147]. The current work has reviewed existing literature and does not include new data, limiting the analysis's depth. The results and findings of this review are constrained by the diversity in the study population and the relatively short follow-up of the reviewed research [148, 149]. Larger and more diverse cohort studies with extended follow-ups are needed to strengthen future investigations' applicability and reliability.

### 4.3 Study Strength

Earlier work in the field had discussed LC therapeutic strategies. A key novelty of the current review is integrating available therapeutic options, from pharmacological and non-pharmacological to innovative and experimental approaches. By exploring cutting-edge approaches in immunomodulators to regenerative medicine and targeted molecular intervention, we aim to reshape LC treatment. Highlighting unresolved gaps and challenges, we emphasize the need for personalized therapeutic strategies and new biomarkers for improved outcomes.

The insight reached will make a clear road map for future research in LC management and provide valuable resources to physicians and policymakers that can guide future directions.

## CONCLUSION

Long COVID has become a major public health concern. The necessity for targeted research to identify its destructive influence on human health is highlighted by the apparent disproportion impact on women, unvaccinated individuals, and those with health co-morbidities. Underscoring the need for focused research to unveil its unique effect on human health.

A comprehensive and critical evaluation of therapeutic strategies for LC was made to improve the current understanding and facilitate the development of effective management strategies for LC cases. Non-pharmacological approaches were endorsed first, showing variable degrees of efficacy, highlighting individual variability and the need for larger, more robust trials.

On the other hand, pharmacological agents showed promising results, but they require further validation through more extended studies to assess their efficacy and safety in the long term.

Many areas of LC are still unknown; ongoing monitoring of LC cases is recommended alongside allocating appropriate community and healthcare resources to address the Long COVID evolving challenge. Given the evolving nature of LC, future research should explore how the emerging SARS-CoV-2 variants, such as Omicron and Delta, influence the disease progression and therapeutic variances. In addition, extended follow-up of LC cases and allocating health resources is vital to address this ongoing challenge. Future studies are needed to refine therapeutic strategies to improve patient outcomes.

## Figures and Tables

**Fig. (1):**
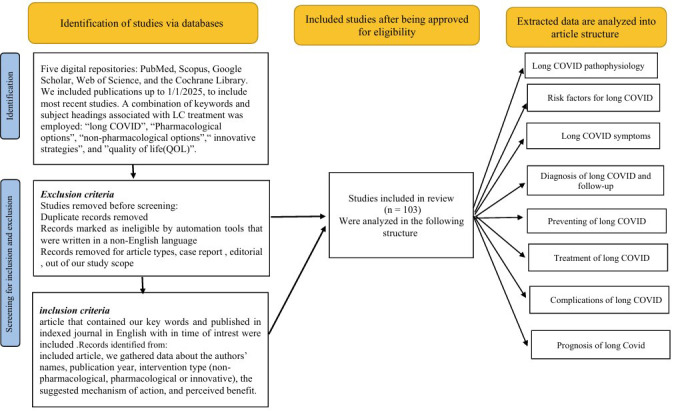
Study flowchart and main review structure.

**Table 1:** Symptoms of long COVID are based on the most frequently reported symptoms and supporting references.

No.	**Symptoms**	**Reported Incidence**	**Description**	**References**
1	Fatigue	Up to 95%	It has been reported for months among patients	Jeong & Kim, 2024 [41]
2	Respiratory complaints in long COVID	Up to 81%	Chronic cough and shortness of breath	Beurnier *et al*, 2023 [42]
3	Cardiovascular symptoms	Frequent	• Fatigue (up to 40%) • Chest pain (up to 20%) • Palpitation (up to 13%) • Dyspnea (up to 37%) • Other, less specific symptoms include Edema, cough, postural orthostatic tachycardia syndrome (POTS), and sweating	Kusumawardhani *et al. *2023 [43]
4	Impaired cognition	Up to 80%	People suffer from memory loss, deficiency in executive functions, and attention. They are collectively called “brain fog”.	Liu *et al.*, 2024 [44]
5	Neurological symptoms	75-60%	They range from insomnia and headache, which negatively affect QOL and everyday performance.	Parums, 2024 [45]
6	Diverse sensory loss	Frequent	Losing sense of taste or smell, and sometimes there are GIT issues	Sharma *et al.*, 2024 [46]
7	Symptoms clusters	Not specified	A collection of symptoms was noticed of many systems: cognitive, Fatigue and pain, respiratory, GIT, dermatology, arthralgia, myalgia, sensory, and emotional clusters.	O'Mahony *et al.*; 2022 [47]
8	Poor QOL	Not specified	Severe physical and mental symptoms inversely impact patients' QOL	Antar & Cox, 2024 [48]

**Abbreviations:** postural orthostatic tachycardia syndrome (POTS); Quality of life (QOL), gastrointestinal tract (GIT).

**Table 2:** Non pharmacological therapeutic strategy for long COVID.

**Methods**	**Subtypes and Mechanism of Action**	**Efficacy in Long COVID**	**Advantages and Drawbacks**	**References**
Exercise-based interventions	Pilates, resistance exercise, telerehabilitation	Improve exercise tolerance, QOL, fatigue; enhance pulmonary fitness, psychological well-being, and improve sleep & cognitive impairment. It is 1^st^ line in addressing POTS	Supporting evidence is limited, yet the efficacy is promising	[71-73]
Neuromodulation [ex. Transcutaneous Electrical Nerve Stimulation; Transcranial Magnetic Stimulation	• It modulates neural pathways to relieve pain, fatigue, and brain fog. • Tested in POTS	Symptomatic relief	The supporting evidence regarding effectiveness is not strong	[74, 75]
Behavioral therapy, cognitive behavioral therapy (CBT)	Beneficial in addressing psychological causes of fatigue & sleep disturbances	Help in developing a coping strategy	Do not address the underlying problem	[76, 77]
Lifestyle modification	Promoting a healthy life *via* better diet, sleep, and stress management	• It showed benefits in overall health and QOL. • Useful in short-term symptom control	Efficacy in the long term needs more evidence. It needs patient adherence	[78, 79]
Wait and see the strategy.	Gives the body time for natural recovery from fatigue and viremia.	Granting sick leaves to affected patients.	It is a passive approach and does not improve QOL.	[80]
Music therapy	• Enhances psychological well-being, improves moods • Alleviates anxiety & sleep disorders.	It was beneficial in symptomatic relief.	The supporting evidence regarding effectiveness is limited	[81]
Acupuncture	Reduces stress, pain, and inflammation	The benefits were reported in terms of physical and psychological symptoms.	The supporting evidence regarding effectiveness is promising	[82]
Hyperbolic oxygen	Reduces the effect of hypoxia on the affected tissue	It was beneficial in LC-related fatigue and brain fog given in 10 sessions	There were significant improvements among treated cases	[83]
Complementary and alternative medicine (CAM) Ex: yoga & aquatic therapy	• Used to manage GIT symptoms *via* an anti-inflammatory effect • Used in addressing fatigue & sleep disturbances	It was effective in controlling nausea and diarrhea. Holistic improvement in mental and physical health.	The supporting evidence regarding effectiveness is not strong	[84]
Nutrients Vitamin D, Omega-3 fatty acids	It exerts anti-inflammatory action and supports immunity	By supporting overall well-being, it promotes healthy recovery It was suggested for POTS cases	Requires more research regarding its efficacy.	[85, 86]
Probiotics Lacto*bacillus, Bifidobacterium*	• Maintain a healthy gut ecosystem, modulate immune function • Reduce oxidative stress	• Beneficial in GIT symptoms, reduces systemic inflammation • Of benefit in psychological symptoms of LC	• Efficacy needs to be validated. • The underlying mechanism that improves LC is uncertain; there is variation and inconsistency in the results reached • There is a potential risk of infection, especially among immune-depressed cases.	[87, 88]

**Abbreviations:** POTS: Postural orthostatic Tachycardia Syndrome.

**Table 3:** Pharmacological Therapeutic Strategies for Long COVID.

**Therapeutic Intervention**	**Example**	**Mechanism of Action**	**Perceived Benefit**	**Efficacy and/ or SE**	**Reference**
Antiviral drugs	Remdesivir	Reduced viral replication	• Reduction of viral load thus reduces infection severity. • It reduces viral shedding.	Using them in acute infection reduces the LC risk by 27% and reduces mortality by 30%	[89]
Respiratory symptoms	• Inhaler • Antihistamines drugs • Corticosteroid (Dexamethasone) • Bronchodilators, cough suppressors • Topical steroids nintedanib or pirfenidone	• Antihistamines showed lower levels of CD4^+^ and CD8^+^ T-cell counts • Steroid cases showed reduced inflammation and cytokine levels • Alleviate cough and enhance airway patency.	• Useful in dyspnea, cough, and respiratory symptoms • Topical steroids were recommended for olfactory dysfunction	• Chronic antihistamines: use and Steroids. Long-term use causes side effects. • Insight: The steroid group did not show significant changes in medical response vs. those who did not take it • The antihistamine group showed better clinical response *vs* those who did not.	[55, 75, 90, 91]
Anti-inflammatory Agents	Non-steroidal anti-inflammatory drugs (NSAIDs)	Reduces pain & inflammation.	• Control musculoskeletal pain and headache attacks. • Relieves inflammatory symptoms	Symptomatic relief only.	[92, 93]
Antidepressants	Selective Serotonin Reuptake Inhibitors (SSRIs)	Modulate neurotransmitters to reduce anxiety and depression	• Beneficial in cases with psychiatric symptoms, as it improves mental health • In refractory headache cases. • It is helpful in reducing fatigue.	It requires monitoring of side effects in long-term use.	[94, 95]
Cardiovascular drugs	• Beta-blockers • Anticoagulant	Manges arrhythmia & reduces thrombosis risk	Rectify CVS dynamics and reduce thrombosis risk	The drugs are given on an individual basis and have side effects like bleeding and bradycardia.	[43]
Neurological drugs	Gabapentin	• Improves cognitive difficulties, *i.e.*, brain fog • reduces nerve pain	Beneficial in neuropathic pain	Its efficacy is limited, and sometimes it was conflicted with anticoagulants.	[96]
Suggested use of theoretical application	Hydroxychloroquine	• Immunomodulatory, anti-inflammatory drug • Has anti-thrombotic action	Suggested use in reducing overall immunological symptoms and reducing thrombosis risk.	Drug role in COVID infection was inconsistent, and their role in LC was theoretical and needed validation.	[97, 98]

**Table 4:** Experimental and innovative therapeutic strategies in long COVID.

**Category**	**Drugs or Intervention**	**Mechanism of Action**	**Special Indication for Use**	**Perceived Benefit**	**References**
Immunoglobulin (IG) Experimental	Intravenous IG	modulate immune response by reducing inflammation	Used for immunological, autoimmune, and inflammatory complications	It needs specialized centers, and it has high costs	[99]
Cytokine inhibitors Experimental	Interleukin receptor antagonists, Janus kinase inhibitors [Ruxolitinib & Baricitinib]	By targeting cytokine pathways, it modulates inflammation	Lowers cytokine storm and associated severe COVID symptoms.	Most of the studies focused on its role in acute infection and short-term complications. It requires more research regarding its safety and efficacy in long COVID.	[100, 101]
Suggested use of an anti-diabetic drug Experimental	Metformin	It exhibits anti-inflammatory and immunomodulatory properties and reduces thrombosis risk.	Especially for fatigue, pain, and post-exertion malaise	Its use reduces LC risk from 40-60%; the supporting evidence was strong.	[102]
Therapeutic apheresis Innovative	Patients underwent two sessions of apheresis aiming to clear up inflammatory mediators in the serum.	It was effective in alleviating neurotransmitter cytokine and normalizing ESR and fibrinogen.	It was suggested as a therapeutic and prognostic biomarker for LC cases	There was a 70% improvement in the long-term symptoms among affected cases, especially for fatigue and post-exertional malaise	[103]
Immunologically based intervention Innovative	Monoclonal antibodies	It has a direct effect on the virus and reduces its persistence	Useful in alleviating neurological symptoms (autonomic dysfunction) & addressing immune regulation by targeting specific pathways	They have a high cost, and their efficacy in LC is under further validation.	[104, 105]
Neuromodulator & anti- inflammatory Innovative	Ibudilast: Neuromodulator Anakinra reduces IL-1	The suggested mechanism is to reduce cytokine production	The tested outcome was improving physical score, fatigue, QOL, brain fog, and reducing mortality	Their RCT was not over yet	[106]
Stem cell therapy Innovative	Stem cells of the umbilical cord	Promotes tissue repair, regeneration, & modulates immune response in terms of T-cells and cytokines (IL-6)	Cases showed good results on patchy lung fibrotic changes control depression, sleep disturbances	Their efficacy was promising, with no major side effects reported. It still needs further validation.	[107]
Adjunctive therapy Innovative	Melatonin	Anti-inflammatory & immune modulation, neuroprotective effects and sleep regulation has theoretical benefits based on NRF2 pathway activation.	It reduces depression, fatigue, and insomnia. It was 2 times as effective as antiviral in reducing inflammatory biomarkers triggered by COVID.	These are perceived benefits in acute covid; however, they are currently no reliable trial in LC	[108, 109]

**Table 5:** The time lag for Long COVID symptoms to resolve.

**Symptom**	**Time to Resolve**	**References**
Impaired memory and lack of concentration	Up to 2 months	[10, 117]
Chest pain	Up to 2-3 months	[10, 118]
Cough	Within 3 months	[10, 119]
Psychological symptoms	More than 6 months	[10, 120]
Fatigue, chest pain, dyspnea	Up to 1 year	[10, 121]
Cognitive impairment	Up to 1 year	[10, 122]

## LIST OF ABBREVIATIONS

LC: Long COVID-19
QOL: Quality of Life
BMI: Body Mass Index
NP: Non-pharmacological
PASC: Post-acute Sequelae of SARS-CoV-2 Infection
CNS: Central Nervous System
OR: Odds Ratio
POTS: Postural Orthostatic Tachycardia Syndrome
GIT: Gastrointestinal
LDH: Lactate Dehydrogenase
CXR: Chest X-ray
CT: Computerized CT scan
ICU: Intensive Care Unit

## References

[1] Ngongolo K. (2023). Impacts, mitigation strategies of COVID-19 on human well-being in Africa: A brief review.. Soc. Sci. Humanit. Open.

[2] Edwards F., Hamilton F.W. (2023). Impact of COVID-19 vaccination on long covid.. BMJ Med..

[3] Sohrabi C., Mathew G., Franchi T., Kerwan A., Griffin M., Soleil C Del Mundo J., Ali S.A., Agha M., Agha R. (2021). Impact of the coronavirus (COVID-19) pandemic on scientific research and implications for clinical academic training – A review.. Int. J. Surg..

[4] Alkodaymi M.S., Omrani O.A., Ashraf N., Shaar B.A., Almamlouk R., Riaz M., Obeidat M., Obeidat Y., Gerberi D., Taha R.M., Kashour Z., Kashour T., Berbari E.F., Alkattan K., Tleyjeh I.M. (2022). Prevalence of post-acute COVID-19 syndrome symptoms at different follow-up periods: A systematic review and meta-analysis.. Clin. Microbiol. Infect..

[5] O’Mahoney L.L., Routen A., Gillies C., Ekezie W., Welford A., Zhang A., Karamchandani U., Simms-Williams N., Cassambai S., Ardavani A., Wilkinson T.J., Hawthorne G., Curtis F., Kingsnorth A.P., Almaqhawi A., Ward T., Ayoubkhani D., Banerjee A., Calvert M., Shafran R., Stephenson T., Sterne J., Ward H., Evans R.A., Zaccardi F., Wright S., Khunti K. (2023). The prevalence and long-term health effects of Long COVID among hospitalised and non-hospitalised populations: A systematic review and meta-analysis.. EClinicalMedicine.

[6] Healey Q., Sheikh A., Daines L., Vasileiou E. (2022). Symptoms and signs of long COVID: A rapid review and meta-analysis.. J. Glob. Health.

[7] (2021). A clinical case definition of post COVID-19 condition by a Delphi consensus.. https://www.who.int/publications/i/item/WHO-2019-nCoV-Post_COVID-19_condition-Clinical_case_definition-2021.1.

[8] Notarte K.I., de Oliveira M.H.S., Peligro P.J., Velasco J.V., Macaranas I., Ver A.T., Pangilinan F.C., Pastrana A., Goldrich N., Kavteladze D., Gellaco M.M.L., Liu J., Lippi G., Henry B.M., Fernández-de-las-Peñas C. (2022). Age, sex and previous comorbidities as risk factors not associated with SARS-CoV-2 infection for long COVID-19: A systematic review and meta-analysis.. J. Clin. Med..

[9] Muthuka J.K., Nzioki J.M., Kelly J.O., Musangi E.N., Chebungei L.C., Nabaweesi R., Kiptoo M.K. (2024). Prevalence and predictors of long COVID-19 and the average time to diagnosis in the general population: A systematic review, meta-analysis and meta-regression.. COVID.

[10] Huerne K., Filion K.B., Grad R., Ernst P., Gershon A.S., Eisenberg M.J. (2023). Epidemiological and clinical perspectives of long COVID syndrome.. Am. J. Med. Open.

[11] Romero-Ibarguengoitia M.E., Rodríguez-Torres J.F., Garza-Silva A., Rivera-Cavazos A., Morales-Rodriguez D.P., Hurtado-Cabrera M., Kalife-Assad R., Villarreal-Parra D., Loose-Esparza A., Gutiérrez-Arias J.J., Mata-Porras Y.G., Ojeda-Salazar D.A., Sanz-Sánchez M.A., González-Cantú A., Azzolini E., Rescigno M. (2024). Association of vaccine status, reinfections, and risk factors with Long COVID syndrome.. Sci. Rep..

[12] Adnan A. (2022). New-onset diabetic ketoacidosis precipitated by COVID-19 in children: A case report.. Kirkuk J. Med. Sci..

[13] Iqbal F.M., Lam K., Sounderajah V., Clarke J.M., Ashrafian H., Darzi A. (2021). Characteristics and predictors of acute and chronic post-COVID syndrome: A systematic review and meta-analysis.. EClinicalMedicine.

[14] Su Y., Yuan D., Chen D.G., Ng R.H., Wang K., Choi J., Li S., Hong S., Zhang R., Xie J., Kornilov S.A., Scherler K., Pavlovitch-Bedzyk A.J., Dong S., Lausted C., Lee I., Fallen S., Dai C.L., Baloni P., Smith B., Duvvuri V.R., Anderson K.G., Li J., Yang F., Duncombe C.J., McCulloch D.J., Rostomily C., Troisch P., Zhou J., Mackay S., DeGottardi Q., May D.H., Taniguchi R., Gittelman R.M., Klinger M., Snyder T.M., Roper R., Wojciechowska G., Murray K., Edmark R., Evans S., Jones L., Zhou Y., Rowen L., Liu R., Chour W., Algren H.A., Berrington W.R., Wallick J.A., Cochran R.A., Micikas M.E., Wrin T., Petropoulos C.J., Cole H.R., Fischer T.D., Wei W., Hoon D.S.B., Price N.D., Subramanian N., Hill J.A., Hadlock J., Magis A.T., Ribas A., Lanier L.L., Boyd S.D., Bluestone J.A., Chu H., Hood L., Gottardo R., Greenberg P.D., Davis M.M., Goldman J.D., Heath J.R. (2022). Multiple early factors anticipate post-acute COVID-19 sequelae.. Cell.

[15] Opsteen S., Files J.K., Fram T., Erdmann N. (2023). The role of immune activation and antigen persistence in acute and long COVID.. J. Investig. Med..

[16] Mihai C.M., Chisnoiu T., Balasa A.L., Frecus C.E., Mihai L., Pantazi A.C. (2023). Clinical characteristics and laboratory findings in children with multisystem inflammatory syndrome (MIS-C)—A retrospective study of a tertiary care center from Constanta, Romania.. Healthcare.

[17] Akram N.N., Ibrahim B.A., Ali S.M., Nori W. (2022). Clinical and laboratory characteristics of children with neurological presentations of COVID-19: A single-center experience.. J. Med. Life.

[18] Georgieva E., Ananiev J., Yovchev Y., Arabadzhiev G., Abrashev H., Abrasheva D., Atanasov V., Kostandieva R., Mitev M., Petkova-Parlapanska K., Karamalakova Y., Koleva-Korkelia I., Tsoneva V., Nikolova G. (2023). COVID-19 complications: Oxidative stress, inflammation, and mitochondrial and endothelial dysfunction.. Int. J. Mol. Sci..

[19] Smadja D.M., Mentzer S.J., Fontenay M., Laffan M.A., Ackermann M., Helms J., Jonigk D., Chocron R., Pier G.B., Gendron N., Pons S., Diehl J.L., Margadant C., Guerin C., Huijbers E.J.M., Philippe A., Chapuis N., Nowak-Sliwinska P., Karagiannidis C., Sanchez O., Kümpers P., Skurnik D., Randi A.M., Griffioen A.W. (2021). COVID-19 is a systemic vascular hemopathy: Insight for mechanistic and clinical aspects.. Angiogenesis.

[20] Jammoul M., Naddour J., Madi A., Reslan M.A., Hatoum F., Zeineddine J., Abou-Kheir W., Lawand N. (2023). Investigating the possible mechanisms of autonomic dysfunction post-COVID-19.. Auton. Neurosci..

[21] Goldstein D.S. (2024). Post-COVID dysautonomias: What we know and (mainly) what we don’t know.. Nat. Rev. Neurol..

[22] Leitner B.P., Joseph P., Quast A.F., Ramirez M.A., Heerdt P.M., Villalobos J.G., Singh I. (2024). The metabolic and physiologic impairments underlying long COVID associated exercise intolerance.. Pulm. Circ..

[23] Zhang D., Zhou Y., Ma Y., Chen P., Tang J., Yang B., Li H., Liang M., Xue Y., Liu Y., Zhang J., Wang X. (2023). Gut microbiota dysbiosis correlates with long COVID-19 at one-year after discharge.. J. Korean Med. Sci..

[24] Bai F., Tomasoni D., Falcinella C., Barbanotti D., Castoldi R., Mulè G., Augello M., Mondatore D., Allegrini M., Cona A., Tesoro D., Tagliaferri G., Viganò O., Suardi E., Tincati C., Beringheli T., Varisco B., Battistini C.L., Piscopo K., Vegni E., Tavelli A., Terzoni S., Marchetti G., Monforte A.A. (2022). Female gender is associated with long COVID syndrome: A prospective cohort study.. Clin. Microbiol. Infect..

[25] Krüger S. (2023). Long-COVID: Vaccination and early antiviral therapy reduce the risk.. Compass Pneumol.

[26] Atchison C.J., Davies B., Cooper E., Lound A., Whitaker M., Hampshire A., Azor A., Donnelly C.A., Chadeau-Hyam M., Cooke G.S., Ward H., Elliott P. (2023). Long-term health impacts of COVID-19 among 242,712 adults in England.. Nat. Commun..

[27] Akram N.N., Nori W., Al Qaissi K.W., Abdulrahman Hadi B.A. (2021). Multi-systemic inflammatory syndrome in childhood (MIS-C): A review article.. J. Pak. Med. Assoc..

[28] Bridger Staatz C., Bann D., Ploubidis G.B., Goodman A., Silverwood R.J. (2023). Age of first overweight and obesity, COVID-19 and long COVID in two british birth cohorts.. J. Epidemiol. Glob. Health.

[29] Tsampasian V., Elghazaly H., Chattopadhyay R., Debski M., Naing T.K.P., Garg P., Clark A., Ntatsaki E., Vassiliou V.S. (2023). Risk factors associated with post−COVID-19 condition.. JAMA Intern. Med..

[30] Song Z., Giuriato M. (2023). Demographic and clinical factors associated with long COVID.. Health Aff..

[31] Nadhmi S. (2021). The manifestation of COVID-19 virus in children in Kirkuk city.. Kirkuk J Med Sci.

[32] Costea D., Serbanescu L., Badiu D., Ardeleanu V., Branescu C., Zgura A., Costea A. (2022). Pain management in the right iliac fossa during the COVID-19 pandemic.. J. Mind Med. Sci..

[33] Ceban F., Kulzhabayeva D., Rodrigues N.B., Di Vincenzo J.D., Gill H., Subramaniapillai M., Lui L.M.W., Cao B., Mansur R.B., Ho R.C., Burke M.J., Rhee T.G., Rosenblat J.D., McIntyre R.S. (2023). COVID-19 vaccination for the prevention and treatment of long COVID: A systematic review and meta-analysis.. Brain Behav. Immun..

[34] Nori W., Ghani Zghair M.A. (2022). Omicron targets upper airways in pediatrics, elderly and unvaccinated population.. World J. Clin. Cases.

[35] Fuller T., Flores Mamani R., Ferreira Pinto Santos H., Melo Espíndola O., Guaraldo L., Lopes Melo C., Borges Da Silva M.F., Amaral Calvet G., Soares Bastos L., Carvalho M.S., Brasil P. (2024). Sex, vaccination status, and comorbidities influence long COVID persistence.. J. Infect. Public Health.

[36] Jeffrey K., Hammersley V., Maini R., Crawford A., Woolford L., Batchelor A., Weatherill D., White C., Millington T., Kerr R., Basetti S., Macdonald C., Quint J.K., Kerr S., Shah S.A., Kurdi A., Simpson C.R., Katikireddi S.V., Rudan I., Robertson C., Ritchie L., Sheikh A., Daines L. (2024). Deriving and validating a risk prediction model for long COVID: A population-based, retrospective cohort study in Scotland.. J. R. Soc. Med..

[37] Azzam A., Khaled H., Refaey N., Mohsen S., El-Emam O.A., Dawood N., Ahmed H.A., Soliman O.A., Mostafa S., Ramadan H., Mosa M., Elmowafy A.O.I., Rizk S.M.A., Zaki A., Hussien M., Ahmed A., Ezzat A.A., Hassan F.E. (2024). The burden of persistent symptoms after COVID-19 (long COVID): A meta-analysis of controlled studies in children and adults.. Virol. J..

[38] Davis H.E., McCorkell L., Vogel J.M., Topol E.J. (2023). Long COVID: Major findings, mechanisms and recommendations.. Nat. Rev. Microbiol..

[39] Ray J.G., Schull M.J., Vermeulen M.J., Park A.L. (2021). Association between abo and rh blood groups and SARS-COV-2 infection or severe COVID-19 illness.. Ann. Intern. Med..

[40] Rabiee Rad M., Abbasi M., Salimian E., Norouzi M., Emamjomeh A., Haghighatdoost F., Mahmoudi S., Najafian J., Masoudi S., Ghasempour Dabaghi G., Mohammadifard N., Sarrafzadegan N. (2024). Baseline socioeconomic status predicting post-COVID-19 symptoms: Results from Isfahan COVID Cohort (ICC) study.. Prev. Med. Rep..

[41] Jeong Y.K., Kim H.Y. (2024). Symptom clusters and quality of life in people with long COVID: A cross-sectional online survey.. J. Korean Acad. Fundam. Nurs..

[42] Beurnier A., Savale L., Jaïs X., Colle R., Pham T., Morin L., Bulifon S., Noël N., Boucly A., Delbarre B., Ebstein N., Figueiredo S., Gasnier M., Harrois A., Jutant E.M., Jevnikar M., Keddache S., Lecoq A.L., Meyrignac O., Parent F., Pichon J., Preda M., Roche A., Seferian A., Bellin M.F., Gille T., Corruble E., Sitbon O., Becquemont L., Monnet X., Humbert M., Montani D., Morin L., Savale L., Pham T., Colle R., Figueiredo S., Harrois A., Gasnier M., Lecoq A-L., Meyrignac O., Noel N., Baudry E., Bellin M-F., Beurnier A., Choucha W., Corruble E., Dortet L., Hardy-Leger I., Radiguer F., Sportouch S., Verny C., Wyplosz B., Zaidan M., Becquemont L., Montani D., Monnet X. (2023). Functional respiratory complaints among COVID-19 survivors: A prospective cohort study.. ERJ Open Res..

[43] Kusumawardhani N.Y., Putra I.C.S., Kamarullah W., Afrianti R., Pramudyo M., Iqbal M., Prameswari H.S., Achmad C., Tiksnadi B.B., Akbar M.R. (2023). Cardiovascular disease in post-acute COVID-19 syndrome: A comprehensive review of pathophysiology and diagnosis approach.. Rev. Cardiovasc. Med..

[44] Liu E.N., Yang J.H., Patel L., Arora J., Gooding A., Ellis R., Graves J.S. (2023). Longitudinal analysis and treatment of neuropsychiatric symptoms in post-acute sequelae of COVID-19.. J. Neurol..

[45] Parums D.V. (2024). Long COVID or post-acute sequelae of SARS-CoV-2 infection (PASC) and the urgent need to identify diagnostic biomarkers and risk factors.. Med. Sci. Monit..

[46] Sharma S.K., Mohan A., Upadhyay V. (2024). Long COVID syndrome: An unfolding enigma.. Indian J. Med. Res..

[47] O’ Mahony L., Buwalda T., Blair M., Forde B., Lunjani N., Ambikan A., Neogi U., Barrett P., Geary E., O’Connor N., Dineen J., Clarke G., Kelleher E., Horgan M., Jackson A., Sadlier C. (2022). Impact of Long COVID on health and quality of life.. HRB Open Res..

[48] Antar A.A.R., Cox A.L. (2024). Translating insights into therapies for Long COVID.. Sci. Transl. Med..

[49] Szewczyk W., Fitzpatrick A.L., Fossou H., Gentile N.L., Sotoodehnia N., Vora S.B., West T.E., Bertolli J., Cope J.R., Lin J.M.S., Unger E.R., Vu Q.M. (2024). Long COVID and recovery from Long COVID: Quality of life impairments and subjective cognitive decline at a median of 2 years after initial infection.. BMC Infect. Dis..

[50] Zaidi A.K., Dehgani-Mobaraki P. (2024). Long Covid.. Prog. Mol. Biol. Transl. Sci..

[51] Tsilingiris D., Vallianou N.G., Karampela I., Christodoulatos G.S., Papavasileiou G., Petropoulou D., Magkos F., Dalamaga M. (2023). Laboratory findings and biomarkers in Long COVID: What do we know so far? Insights into epidemiology, pathogenesis, therapeutic perspectives and challenges.. Int. J. Mol. Sci..

[52] Nori W., Hameed B.H., Thamir A.R., Fadhil A. (2021). COVID-19 in pregnancy: Implication on platelets and blood indices.. Rev. Bras. Ginecol. Obstet..

[53] Lai Y.J., Liu S.H., Manachevakul S., Lee T.A., Kuo C.T., Bello D. (2023). Biomarkers in long COVID-19: A systematic review.. Front. Med..

[54] Klein J., Wood J., Jaycox J.R., Dhodapkar R.M., Lu P., Gehlhausen J.R., Tabachnikova A., Greene K., Tabacof L., Malik A.A., Silva Monteiro V., Silva J., Kamath K., Zhang M., Dhal A., Ott I.M., Valle G., Peña-Hernández M., Mao T., Bhattacharjee B., Takahashi T., Lucas C., Song E., McCarthy D., Breyman E., Tosto-Mancuso J., Dai Y., Perotti E., Akduman K., Tzeng T.J., Xu L., Geraghty A.C., Monje M., Yildirim I., Shon J., Medzhitov R., Lutchmansingh D., Possick J.D., Kaminski N., Omer S.B., Krumholz H.M., Guan L., Dela Cruz C.S., van Dijk D., Ring A.M., Putrino D., Iwasaki A. (2023). Distinguishing features of long COVID identified through immune profiling.. Nature.

[55] Antoniou K.M., Vasarmidi E., Russell A.M., Andrejak C., Crestani B., Delcroix M., Dinh-Xuan A.T., Poletti V., Sverzellati N., Vitacca M., Witzenrath M., Tonia T., Spanevello A. (2022). European respiratory society statement on long COVID follow-up.. Eur. Respir. J..

[56] Abdulqader S.K., Ali S.S.M., Fadhil A.A., Akram N.N., Hassan W.N.M. (2024). Impact of Vaccination Status on Chest CT Findings and Disease Outcomes in COVID-19 Era: A Retrospective Study.. Coronaviruses.

[57] Stoian M., Roman A., Boeriu A., Onișor D., Bandila S.R., Babă D.F., Cocuz I., Niculescu R., Costan A., Laszlo S.Ș., Corău D., Stoian A. (2023). Long-term radiological pulmonary changes in mechanically ventilated patients with respiratory failure due to SARS-CoV-2 infection.. Biomedicines.

[58] Liu K., Zhang W., Yang Y., Zhang J., Li Y., Chen Y. (2020). Respiratory rehabilitation in elderly patients with COVID-19: A randomized controlled study.. Complement. Ther. Clin. Pract..

[59] Rotz S.J., Bhatt N.S., Hamilton B.K., Duncan C., Aljurf M., Atsuta Y., Beebe K., Buchbinder D., Burkhard P., Carpenter P.A., Chaudhri N., Elemary M., Elsawy M., Guilcher G.M.T., Hamad N., Karduss A., Peric Z., Purtill D., Rizzo D., Rodrigues M., Ostriz M.B.R., Salooja N., Schoemans H., Seber A., Sharma A., Srivastava A., Stewart S.K., Baker K.S., Majhail N.S., Phelan R. (2024). International recommendations for screening and preventative practices for long-term survivors of transplantation and cellular therapy: A 2023 update.. Transplant. Cell. Ther..

[60] Gyöngyösi M., Alcaide P., Asselbergs F.W., Brundel B.J.J.M., Camici G.G., Martins P.C., Ferdinandy P., Fontana M., Girao H., Gnecchi M., Gollmann-Tepeköylü C., Kleinbongard P., Krieg T., Madonna R., Paillard M., Pantazis A., Perrino C., Pesce M., Schiattarella G.G., Sluijter J.P.G., Steffens S., Tschöpe C., Van Linthout S., Davidson S.M. (2023). Long COVID and the cardiovascular system—elucidating causes and cellular mechanisms in order to develop targeted diagnostic and therapeutic strategies: A joint scientific statement of the ESC working groups on cellular biology of the heart and myocardial and pericardial diseases.. Cardiovasc. Res..

[61] Machado F.V.C., Meys R., Delbressine J.M., Vaes A.W., Goërtz Y.M.J., van Herck M., Houben-Wilke S., Boon G.J.A.M., Barco S., Burtin C., van ’t Hul A., Posthuma R., Franssen F.M.E., Spies Y., Vijlbrief H., Pitta F., Rezek S.A., Janssen D.J.A., Siegerink B., Klok F.A., Spruit M.A. (2021). Construct validity of the post-COVID-19 functional status scale in adult subjects with COVID-19.. Health Qual. Life Outcomes.

[62] Jacobs L.G., Paleoudis E.G., Bari D.L., Di, Nyirenda T., Friedman T., Gupta A. (2020). Persistence of symptoms and quality of life at 35 days after hospitalization for COVID-19 infection.. PLoS One.

[63] Peluso M.J., Deeks S.G. (2024). Mechanisms of long COVID and the path toward therapeutics.. Cell.

[64] Azzolini E., Levi R., Sarti R., Pozzi C., Mollura M., Mantovani A., Rescigno M. (2022). Association between BNT162b2 vaccination and long COVID after infections not requiring hospitalization in health care workers.. JAMA.

[65] Català M., Mercadé-Besora N., Kolde R., Trinh N.T.H., Roel E., Burn E., Rathod-Mistry T., Kostka K., Man W.Y., Delmestri A., Nordeng H.M.E., Uusküla A., Duarte-Salles T., Prieto-Alhambra D., Jödicke A.M. (2024). The effectiveness of COVID-19 vaccines to prevent long COVID symptoms: Staggered cohort study of data from the UK, Spain, and Estonia.. Lancet Respir. Med..

[66] Domènech-Montoliu S., Puig-Barberà J., Badenes-Marques G., Gil-Fortuño M., Orrico-Sánchez A., Pac-Sa M.R., Perez-Olaso O., Sala-Trull D., Sánchez-Urbano M., Arnedo-Pena A. (2023). Long COVID prevalence and the impact of the third SARS-CoV-2 vaccine dose: A cross-sectional analysis from the third follow-up of the borriana cohort, Valencia, Spain (2020–2022).. Vaccines.

[67] Lotfi H., Mazar M.G., Ei N.M.H., Fahim M., Yazdi N.S. (2023). Vaccination is the most effective and best way to avoid the disease of COVID-19.. Immun. Inflamm. Dis..

[68] Bielecka-Dabrowa A., Sakowicz A., Gryglewska-Wawrzak K., Kapusta J., Banach M., Jankowski P., Chudzik M. (2024). The effect of sex on the risk of long- COVID and cardiovascular complications in healthy patients without comorbidities: Data from a polish long- COVID cardiovascular (PoLoCOV-CVD) study.. J. Clin. Med..

[69] Chandan J.S., Brown K.R., Simms-Williams N., Bashir N.Z., Camaradou J., Heining D., Turner G.M., Rivera S.C., Hotham R., Minhas S., Nirantharakumar K., Sivan M., Khunti K., Raindi D., Marwaha S., Hughes S.E., McMullan C., Marshall T., Calvert M.J., Haroon S., Aiyegbusi O.L., TLC Study (2023). Non-pharmacological therapies for post-viral syndromes, including Long COVID: A systematic review.. Int. J. Environ. Res. Public Health.

[70] Nori W., Kassim M.A.K., Helmi Z.R., Pantazi A.C., Brezeanu D., Brezeanu A.M., Penciu R.C., Serbanescu L. (2023). Non-pharmacological pain management in labor: A systematic review.. J. Clin. Med..

[71] Chandan J.S., Brown K., Simms-Williams N., Camaradou J., Bashir N., Heining D., Aiyegbusi O.L., Turner G., Cruz Rivera S., Hotham R., Nirantharakumar K., Sivan M., Khunti K., Raindi D., Marwaha S., Hughes S.E., McMullan C., Calvert M., Haroon S. (2022). Non-pharmacological therapies for postviral syndromes, including Long COVID: A systematic review and meta-analysis protocol.. BMJ Open.

[72] Fine J.S., Ambrose A.F., Didehbani N., Fleming T.K., Glashan L., Longo M., Merlino A., Ng R., Nora G.J., Rolin S., Silver J.K., Terzic C.M., Verduzco-Gutierrez M., Sampsel S. (2022). Multi-disciplinary collaborative consensus guidance statement on the assessment and treatment of cognitive symptoms in patients with post-acute sequelae of SARS-CoV-2 infection (PASC).. PMR.

[73] Desai A.D., Boursiquot B.C., Melki L., Wan E.Y. (2021). Management of arrhythmias associated with COVID-19.. Curr. Cardiol. Rep..

[74] Knotkova H., Hamani C., Sivanesan E., Le Beuffe M.F.E., Moon J.Y., Cohen S.P., Huntoon M.A. (2021). Neuromodulation for chronic pain.. Lancet.

[75] Seo J.W., Kim S.E., Kim Y., Kim E.J., Kim T., Kim T., Lee S.H., Lee E., Lee J., Seo Y.B., Jeong Y.H., Jung Y.H., Choi Y.J., Song J.Y. (2024). Updated clinical practice guidelines for the diagnosis and management of long COVID.. Infect. Chemother..

[76] Kuut T.A., Müller F., Csorba I., Braamse A., Aldenkamp A., Appelman B., Assmann-Schuilwerve E., Geerlings S.E., Gibney K.B., Kanaan R.A.A., Mooij-Kalverda K., Olde Hartman T.C., Pauëlsen D., Prins M., Slieker K., van Vugt M., Keijmel S.P., Nieuwkerk P., Rovers C.P., Knoop H. (2023). Efficacy of cognitive-behavioral therapy targeting severe fatigue following coronavirus disease 2019: Results of a randomized controlled trial.. Clin. Infect. Dis..

[77] Kassim M.A.K., Pantazi A.C., Nori W., Tuta L.A., Balasa A.L., Mihai C.M., Mihai L., Frecus C.E., Lupu V.V., Lupu A., Andrusca A., Iorga A.M., Litrin R.M., Ion I., Ciciu E., Chirila S.I., Chisnoiu T. (2023). Non-pharmacological interventions for pain management in hemodialysis: A narrative review.. J. Clin. Med..

[78] Zhuang W., Fan Z., Chu Y., Wang H., Yang Y., Wu L., Sun N., Sun G., Shen Y., Lin X., Guo G. (2020). Chinese patent medicines in the treatment of coronavirus disease 2019 (COVID-19) in China.. Front Pharmacol.

[79] Nori W., Akram N.N., Al-Kaabi M.M. (2023). Probiotics in women and pediatrics health: A narrative review.. Al-Anbar Med. J..

[80] Bachmeier B.E., Hölzle S., Gasser M., van den Akker M. (2023). How do German General Practitioners Manage Long-/Post-COVID? A Qualitative Study in Primary Care.. Viruses.

[81] Wang C.C., Lo J., Saunders R., Adama E., Bulsara C., Etherton-Beer C., Yang A.W.H. (2022). Light acupuncture and five-element music therapy for nurses’ mental health and well-being during and post-COVID-19: Protocol for a randomised cross-over feasibility study.. BMJ Open.

[82] Lam W.C., Wei D., Li H., Yao L., Zhang S., Lai M.X.Y., Zheng Y., Yeung J.W.F., Lau A.Y.L., Lyu A., Bian Z., Cheung A.M., Zhong L.L.D. (2024). The use of acupuncture for addressing neurological and neuropsychiatric symptoms in patients with long COVID: A systematic review and meta-analysis.. Front. Neurol..

[83] Robbins T., Gonevski M., Clark C., Baitule S., Sharma K., Magar A. (2021). Hyperbaric oxygen therapy for the treatment of long COVID: Early evaluation of a highly promising intervention.. Clin Med.

[84] Gawey B., Yang J., Bauer B., Song J., Wang X.J. (2023). The use of complementary and alternative medicine for the treatment of gastrointestinal symptoms in Long COVID: A systematic review.. Ther. Adv. Chronic Dis..

[85] Shah K., Varna V.P., Sharma U., Mavalankar D. (2022). Does vitamin D supplementation reduce COVID-19 severity?: A systematic review.. QJM.

[86] Bradbury J., Wilkinson S., Schloss J. (2023). Nutritional support during long COVID: A systematic scoping review.. J. Integr. Complement. Med..

[87] Alenazy M.F., Aljohar H.I., Alruwaili A.R., Daghestani M.H., Alonazi M.A., Labban R.S., El-Ansary A.K., Balto H.A. (2022). Gut microbiota dynamics in relation to long- COVID-19 syndrome: Role of probiotics to combat psychiatric complications.. Metabolites.

[88] Pantazi A.C., Kassim M.A.K., Nori W., Tuta L.A., Mihai C.M., Chisnoiu T., Balasa A.L., Mihai L., Lupu A., Frecus C.E., Lupu V.V., Chirila S.I., Badescu A.G., Hangan L.T., Cambrea S.C. (2023). Clinical perspectives of gut microbiota in patients with chronic kidney disease and end-stage kidney disease: Where do we stand?. Biomedicines.

[89] Choi Y.J., Seo Y.B., Seo J.W., Lee J., Nham E., Seong H., Yoon J.G., Noh J.Y., Cheong H.J., Kim W.J., Kim E.J., Song J.Y. (2023). Effectiveness of anti-viral therapy on long COVID: A systematic review and meta-analysis.. J. Clin. Med..

[90] Goel N., Goyal N., Nagaraja R., Kumar R. (2021). Systemic corticosteroids for management of ‘long-COVID’: An evaluation after 3 months of treatment.. Monaldi Arch. Chest Dis..

[91] Glynne P., Tahmasebi N., Gant V., Gupta R. (2022). Long COVID following mild SARS-CoV-2 infection: Characteristic T cell alterations and response to antihistamines.. J. Investig. Med..

[92] Bonilla H., Peluso M.J., Rodgers K., Aberg J.A., Patterson T.F., Tamburro R., Baizer L., Goldman J.D., Rouphael N., Deitchman A., Fine J., Fontelo P., Kim A.Y., Shaw G., Stratford J., Ceger P., Costantine M.M., Fisher L., O’Brien L., Maughan C., Quigley J.G., Gabbay V., Mohandas S., Williams D., McComsey G.A. (2023). Therapeutic trials for long COVID-19: A call to action from the interventions taskforce of the RECOVER initiative.. Front. Immunol..

[93] Sun G., Lin K., Ai J., Zhang W. (2024). The efficacy of antivirals, corticosteroids, and monoclonal antibodies as acute COVID-19 treatments in reducing the incidence of long COVID: A systematic review and meta-analysis.. Clin. Microbiol. Infect..

[94] Nobile B., Durand M., Olié E., Guillaume S., Molès J.P., Haffen E., Courtet P. (2021). The anti-inflammatory effect of the tricyclic antidepressant clomipramine and its high penetration in the brain might be useful to prevent the psychiatric consequences of SARS-CoV-2 infection.. Front. Pharmacol..

[95] Farahani R.H., Ajam A., Naeini A.R. (2023). Effect of fluvoxamine on preventing neuropsychiatric symptoms of post COVID syndrome in mild to moderate patients, a randomized placebo-controlled double-blind clinical trial.. BMC Infect. Dis..

[96] Stefanou M.I., Palaiodimou L., Bakola E., Smyrnis N., Papadopoulou M., Paraskevas G.P., Rizos E., Boutati E., Grigoriadis N., Krogias C., Giannopoulos S., Tsiodras S., Gaga M., Tsivgoulis G. (2022). Neurological manifestations of long-COVID syndrome: A narrative review.. Ther. Adv. Chronic Dis..

[97] Wang S.S.Y., Xu C. (2023). Hydroxychloroquine: Is there a role in long COVID?. Clin. Rheumatol..

[98] Nori W., Akram N.N., Al-Ani R.M. (2023). Update on hydroxychloroquine use in pregnancy.. World J. Exp. Med..

[99] McCarthy M.W. (2023). Intravenous immunoglobulin as a potential treatment for long COVID.. Expert Opin. Biol. Ther..

[100] Rizk J.G., Kalantar-Zadeh K., Mehra M.R., Lavie C.J., Rizk Y., Forthal D.N. (2020). Pharmaco-immunomodulatory therapy in COVID-19.. Drugs.

[101] Zhang X., Shang L., Fan G., Gu X., Xu J., Wang Y., Huang L., Cao B. (2022). The efficacy and safety of janus kinase inhibitors for patients with COVID-19: A living systematic review and meta-analysis.. Front. Med..

[102] Bramante C.T., Beckman K.B., Mehta T., Karger A.B., Odde D.J., Tignanelli C.J., Buse J.B., Johnson D.M., Watson R.H.B., Daniel J.J., Liebovitz D.M., Nicklas J.M., Cohen K., Puskarich M.A., Belani H.K., Siegel L.K., Klatt N.R., Anderson B., Hartman K.M., Rao V., Hagen A.A., Patel B., Fenno S.L., Avula N., Reddy N.V., Erickson S.M., Fricton R.D., Lee S., Griffiths G., Pullen M.F., Thompson J.L., Sherwood N.E., Murray T.A., Rose M.R., Boulware D.R., Huling J.D., Anderson B., Atwater R.C., Avula N., Beckman K.B., Belani H.K., Boulware D.R., Bramante C.T., Brea J., Broedlow C.A., Buse J.B., Campora P., Challa A., Charles J., Christensen G., Christiansen T., Cohen K., Connelly B., Datta S., Deng N., Dunn A.T., Erickson S.M., Fairbairn F.M., Fenno S.L., Fraser D.J., Fricton R.D., Griffiths G., Hagen A.A., Hartman K.M., Hendrickson A.F., Huling J.D., Ingraham N.E., Jeng A.C., Johnson D.M., Karger A.B., Klatt N.R., Kuehl E.A., LaBar D.D., Lee S., Liebovitz D.M., Lindberg S., Luke D.G., Machicado R., Mohamud Z., Murray T.A., Ngonyama R., Nicklas J.M., Odde D.J., Parrens E., Parra D., Patel B., Proper J.L., Pullen M.F., Puskarich M.A., Rao V., Reddy N.V., Reddy N., Rypka K.J., Saveraid H.G., Seloadji P., Shahriar A., Sherwood N., Siegart J.L., Siegel L.K., Simmons L., Sinelli I., Singh P., Snyder A., Stauffer M.T., Thompson J., Tignanelli C.J., Tople T.L., Tordsen W.J., Watson R.H.B., Wu B., Zaman A., Zolik M.R., Zinkl L. (2024). Favorable antiviral effect of metformin on SARS-CoV-2 viral load in a randomized, placebo-controlled clinical trial of COVID-19.. Clin. Infect. Dis..

[103] Achleitner M., Steenblock C., Dänhardt J., Jarzebska N., Kardashi R., Kanczkowski W., Straube R., Rodionov R.N., Bornstein N., Tselmin S., Kaiser F., Bucher R., Barbir M., Wong M.L., Voit-Bak K., Licinio J., Bornstein S.R. (2023). Clinical improvement of Long-COVID is associated with reduction in autoantibodies, lipids, and inflammation following therapeutic apheresis.. Mol. Psychiatry.

[104] Proal A.D., Aleman S., Bomsel M., Brodin P., Buggert M., Cherry S., Chertow D.S., Davies H.E., Dupont C.L., Deeks S.G., Ely E.W., Fasano A., Freire M., Geng L.N., Griffin D.E., Henrich T.J., Hewitt S.M., Iwasaki A., Krumholz H.M., Locci M., Marconi V.C., Mehandru S., Muller-Trutwin M., Painter M.M., Pretorius E., Price D.A., Putrino D., Qian Y., Roan N.R., Salmon D., Tan G.S., VanElzakker M.B., Wherry E.J., Van Weyenbergh J., Yonker L.M., Peluso M.J. (2025). Targeting the SARS-CoV-2 reservoir in long COVID.. Lancet Infect. Dis..

[105] Cimellaro A., Addesi D., Cavallo M., Spagnolo F., Suraci E., Cordaro R., Spinelli I., Passafaro F., Colosimo M., Pintaudi M., Pintaudi C. (2022). Monoclonal antibodies and antivirals against SARS-CoV-2 reduce the risk of long COVID: A retrospective propensity score-matched case–control study.. Biomedicines.

[106] Baz M., Baker B., Kumar P., Lange U., Livieratos A., Gogos C. (2024). Beyond antivirals: Alternative therapies for long COVID.. Viruses.

[107] Tang Y., Zou X., Liu P., Dai Y., Wang S., Su X., Yu Y., Tang W., Zhou J., Li C., Mei H., Xiao N., Ou Y., Wang J., Lu G., Lin G., Cheng L. (2024). Human umbilical cord-derived mesenchymal stem cell transplantation improves the long COVID.. J. Med. Virol..

[108] Castle R.D., Williams M.A., Bushell W.C., Rindfleisch J.A., Peterson C.T., Marzolf J., Brouwer K., Mills P.J. (2021). Implications for systemic approaches to COVID-19: Effect sizes of remdesivir, tocilizumab, melatonin, vitamin d3, and meditation.. J. Inflamm. Res..

[109] Cardinali D.P., Brown G.M., Pandi-Perumal S.R. (2022). Possible application of melatonin in long COVID.. Biomolecules.

[110] Carlile O., Briggs A., Henderson A.D., Butler-Cole B.F.C., Tazare J., Tomlinson L.A., Marks M., Jit M., Lin L.Y., Bates C., Parry J., Bacon S.C.J., Dillingham I., Dennison W.A., Costello R.E., Walker A.J., Hulme W., Goldacre B., Mehrkar A., MacKenna B., Walker A., Green A., Mehrkar A., Schaffer A., Brown A., Goldacre B., Butler-Cole B., MacKenna B., Morton C., Walters C., Stables C., Cunningham C., Wood C., Andrews C., Evans D., Hickman G., Curtis H., Drysdale H., Dillingham I., Morley J., Massey J., Nab L., Hopcroft L., Fisher L., Bridges L., Wiedemann M., DeVito N., Macdonald O., Inglesby P., Smith R., Croker R., Park R., Higgins R., Bacon S., Davy S., Maude S., O’Dwyer T., Ward T., Speed V., Hulme W., Hart L., Stokes P., Bhaskaran K., Costello R., Cowling T., Douglas I., Eggo R., Evans S., Forbes H., Grieve R., Grint D., Herrett E., Langan S., Mahalingasivam V., Mansfield K., Mathur R., McDonald H., Parker E., Rentsch C., Schultze A., Smeeth L., Tazare J., Tomlinson L., Walker J., Williamson E., Wing K., Wong A., Zheng B., Bates C., Cockburn J., Parry J., Hester F., Harper S., O’Hanlon S., Eavis A., Jarvis R., Avramov D., Griffiths P., Fowles A., Parkes N., Perera R., Harrison D., Khunti K., Sterne J., Quint J., Herrett E., Eggo R.M. (2024). Impact of long COVID on health-related quality-of-life: An OpenSAFELY population cohort study using patient-reported outcome measures (OpenPROMPT).. Lancet Reg. Health Eur..

[111] Sisó-Almirall A., Brito-Zerón P., Conangla Ferrín L., Kostov B., Moragas Moreno A., Mestres J., Sellarès J., Galindo G., Morera R., Basora J., Trilla A., Ramos- Casals M. (2021). Long COVID-19: Proposed primary care clinical guidelines for diagnosis and disease management.. Int. J. Environ. Res. Public Health.

[112] Sapna F.N.U., Deepa F.N.U., Sakshi F.N.U., Sonam F.N.U., Kiran F.N.U., Perkash R.S., Bendari A., Kumar A., Rizvi Y., Suraksha F.N.U., Varrassi G. (2023). Unveiling the mysteries of long COVID syndrome: Exploring the distinct tissue and organ pathologies linked to prolonged COVID-19 symptoms.. Cureus.

[113] Zang C., Hou Y., Schenck E.J., Xu Z., Zhang Y., Xu J., Bian J., Morozyuk D., Khullar D., Nordvig A.S., Shenkman E.A., Rothman R.L., Block J.P., Lyman K., Zhang Y., Varma J., Weiner M.G., Carton T.W., Wang F., Kaushal R. (2024). Identification of risk factors of Long COVID and predictive modeling in the RECOVER EHR cohorts.. Commun. Med..

[114] Maglietta G., Diodati F., Puntoni M., Lazzarelli S., Marcomini B., Patrizi L., Caminiti C. (2022). Prognostic factors for post-COVID-19 syndrome: A systematic review and meta- analysis.. J. Clin. Med..

[115] Mohammedain S.A., Badran S., Elzouki A.Y., Salim H., Chalaby A., Siddiqui M.Y.A., Hussein Y.Y., Rahim H.A., Thalib L., Alam M.F., Al-Badriyeh D., Al- Maadeed S., Doi S.A.R. (2023). Validation of a risk prediction model for COVID-19: The PERIL prospective cohort study.. Future Virol..

[116] Tang C.C., Wu W.W., Ho S.J., Liu W.D., Pan M.Y., Chang S.C., Wang W.S., Yeh Y.C., Chen C.H., Chang J.C. (2025). Clinically significant functional impairments and symptoms in COVID-19 survivors: Empirical research quantitative.. J. Clin. Nurs..

[117] Halpin S.J., McIvor C., Whyatt G., Adams A., Harvey O., McLean L., Walshaw C., Kemp S., Corrado J., Singh R., Collins T., O’Connor R.J., Sivan M. (2021). Postdischarge symptoms and rehabilitation needs in survivors of COVID-19 infection: A cross-sectional evaluation.. J. Med. Virol..

[118] Huang L., Yao Q., Gu X., Wang Q., Ren L., Wang Y., Hu P., Guo L., Liu M., Xu J., Zhang X., Qu Y., Fan Y., Li X., Li C., Yu T., Xia J., Wei M., Chen L., Li Y., Xiao F., Liu D., Wang J., Wang X., Cao B. (2021). 1-year outcomes in hospital survivors with COVID-19: A longitudinal cohort study.. Lancet.

[119] Tenforde M.W., Kim S.S., Lindsell C.J., Billig Rose E., Shapiro N.I., Files D.C., Gibbs K.W., Erickson H.L., Steingrub J.S., Smithline H.A., Gong M.N., Aboodi M.S., Exline M.C., Henning D.J., Wilson J.G., Khan A., Qadir N., Brown S.M., Peltan I.D., Rice T.W., Hager D.N., Ginde A.A., Stubblefield W.B., Patel M.M., Self W.H., Feldstein L.R., Hart K.W., McClellan R., Dorough L., Dzuris N., Griggs E.P., Kassem A.M., Marcet P.L., Ogokeh C.E., Sciarratta C.N., Siddula A., Smith E.R., Wu M.J. (2020). Symptom duration and risk factors for delayed return to usual health among outpatients with COVID-19 in a multistate health care systems network — United States, March–June 2020.. MMWR Morb. Mortal. Wkly. Rep..

[120] Goërtz Y.M.J., Van Herck M., Delbressine J.M., Vaes A.W., Meys R., Machado F.V.C., Houben-Wilke S., Burtin C., Posthuma R., Franssen F.M.E., van Loon N., Hajian B., Spies Y., Vijlbrief H., van ’t Hul A.J., Janssen D.J.A., Spruit M.A. (2020). Persistent symptoms 3 months after a SARS-CoV-2 infection: The post-COVID-19 syndrome?. ERJ Open Res..

[121] Hopkins C., Surda P., Whitehead E., Kumar B.N. (2020). Early recovery following new onset anosmia during the COVID-19 pandemic – An observational cohort study.. J. Otolaryngol. Head Neck Surg..

[122] Carfì A., Bernabei R., Landi F. (2020). Persistent symptoms in patients after acute COVID-19.. JAMA.

[123] Sutherland S. (2023). Long COVID now looks like a neurological disease, helping doctors to focus treatments.. https://www.scientificamerican.com/article/long-covid-now-looks-like-a-neurological-disease-helping-doctors-to-focus-treatments1/.

[124] Rathod N., Kumar S., Chavhan R., Acharya S., Rathod S., Rathod N. (2024). Navigating the long haul: A comprehensive review of long-COVID sequelae, patient impact, pathogenesis, and management.. Cureus.

[125] Reiss A.B., Greene C., Dayaramani C., Rauchman S.H., Stecker M.M., De Leon J., Pinkhasov A. (2023). Long COVID, the brain, nerves, and cognitive function.. Neurol. Int..

[126] Whitaker-Hardin B., McGregor K.M., Uswatte G., Lokken K. (2025). A narrative review of the efficacy of long COVID interventions on brain fog, processing speed, and other related cognitive outcomes.. Biomedicines.

[127] Sousa N.M.F., Maranhão A.C.P.F., Braga L.W. (2024). Cognitive impairment and neuropsychiatric symptoms among individuals with history of symptomatic SARS-CoV-2 infection: A retrospective longitudinal study.. Dement. Neuropsychol..

[128] Fashkhami M.N., Mohammadi S.P. (2024). The effectiveness of cognitive rehabilitation in improving working memory and attention in acute COVID-19 survivors.. Shahroud. J. Med. Sci..

[129] Dong Y., Ritto A.P., Damiano R.F., Coli A.G., Hadade R., Rocca C.C.A., Serafim A.P., Guedes B.F., Nitrini R., Imamura M., Forlenza O.V., Busatto Filho G. (2024). Memory complaints after COVID-19: A potential indicator of primary cognitive impairment or a correlate of psychiatric symptoms?. Transl. Psychiatry.

[130] Talkington G.M., Kolluru P., Gressett T.E., Ismael S., Meenakshi U., Acquarone M., Solch-Ottaiano R.J., White A., Ouvrier B., Paré K., Parker N., Watters A., Siddeeque N., Sullivan B., Ganguli N., Calero-Hernandez V., Hall G., Longo M., Bix G.J. (2025). Neurological sequelae of long COVID: A comprehensive review of diagnostic imaging, underlying mechanisms, and potential therapeutics.. Front. Neurol..

[131] Forero K., Buqaileh R., Sunderman C., AbouAlaiwi W. (2023). COVID-19 and neurological manifestations.. Brain Sci..

[132] Jarrott B., Head R., Pringle K.G., Lumbers E.R., Martin J.H. (2022). “LONG COVID”—A hypothesis for understanding the biological basis and pharmacological treatment strategy.. Pharmacol. Res. Perspect..

[133] Aldhawyan A.F. (2024). Evaluating the predictors of persistent long COVID symptoms and their severity in COVID-19 survivors 1 year after infection.. J. Prim. Care Community Health.

[134] Gennaro F.D., Veronese N., Segala F.V., Frallonardo L., Guido G., Cormio M., Romita G., Parisi A., Marrone E., Ciuppa M.E., Carrubba A.L., Carruba L., Licata A., Cavallaro G., Pagliuso V., Maino T., Lollo S., Latino L., Solimeo L.T., Ianniello A., Montalbò D., Bavaro D.F., Fiorella M.L., Barbagallo M., Saracino A. (2024). Protective role of vaccination on the development of long COVID: Data from a large, multicenter, prospective cohort study.. BMC Infect. Dis..

[135] McKibbin W., Fernando R. (2023). The global economic impacts of the COVID-19 pandemic.. Econ. Model..

[136] Liu Y., Gu X., Li H., Zhang H., Xu J. (2023). Mechanisms of long COVID: An updated review.. Chin. Med. J. Pulm. Crit. Care Med..

[137] Sookaromdee P., Wiwanitkit V. (2024). Impact of COVID-19 pandemic on vertical transmission of HIV in an endemic area in indochina: A preliminary report.. Mustansiriya Med. J..

[138] Serapide F., Talarico M., Rotundo S., Pascale V., Serraino R., Trecarichi E.M., Russo A. (2024). Lights and shadows of long COVID: Are latent infections the real hidden enemy?. J. Clin. Med..

[139] Phillips S., Williams M.A. (2024). Re-framing Long COVID as central nervous system dysfunction.. Science Insights.

[140] Wong A.C., Devason A.S., Umana I.C., Cox T.O., Dohnalová L., Litichevskiy L., Perla J., Lundgren P., Etwebi Z., Izzo L.T., Kim J., Tetlak M., Descamps H.C., Park S.L., Wisser S., McKnight A.D., Pardy R.D., Kim J., Blank N., Patel S., Thum K., Mason S., Beltra J.C., Michieletto M.F., Ngiow S.F., Miller B.M., Liou M.J., Madhu B., Dmitrieva-Posocco O., Huber A.S., Hewins P., Petucci C., Chu C.P., Baraniecki-Zwil G., Giron L.B., Baxter A.E., Greenplate A.R., Kearns C., Montone K., Litzky L.A., Feldman M., Henao-Mejia J., Striepen B., Ramage H., Jurado K.A., Wellen K.E., O’Doherty U., Abdel-Mohsen M., Landay A.L., Keshavarzian A., Henrich T.J., Deeks S.G., Peluso M.J., Meyer N.J., Wherry E.J., Abramoff B.A., Cherry S., Thaiss C.A., Levy M. (2023). Serotonin reduction in post-acute sequelae of viral infection.. Cell.

[141] Joffe A.R., Elliott A. (2023). Long COVID as a functional somatic symptom disorder caused by abnormally precise prior expectations during Bayesian perceptual processing: A new hypothesis and implications for pandemic response.. SAGE Open Med..

[142] Celiker F.B., Kanat A., Turan A., Beyazal M., Burakgazi G., Hursoy N., Gundogdu H., Polat H.B. (2023). Could the cerebral involvement of COVID-19 disease be related to microstructural changes that are not reflected in conventional MRI images?. Neurol. India.

[143] Ali E.A., Salman D.A., Nori W. (2022). C-reactive protein in elderly and pregnant COVID-19 cases: A new role for an old marker.. Mustansiriya Med. J..

[144] Li G., Hilgenfeld R., Whitley R., De Clercq E. (2023). Therapeutic strategies for COVID-19: Progress and lessons learned.. Nat. Rev. Drug Discov..

[145] Nori W., Fadhil A., Jaafar Z.A.A. (2024). The impact of the coronavirus pandemic on telemedicine evolution in obstetrical care during COVID-19.. Coronaviruses.

[146] Catania V., Rundo F., Panerai S., Ferri R. (2023). Virtual reality for the rehabilitation of acquired cognitive disorders: A narrative review.. Bioengineering.

[147] Naidu S.B., Shah A.J., Saigal A., Smith C., Brill S.E., Goldring J., Hurst J.R., Jarvis H., Lipman M., Mandal S. (2021). The high mental health burden of “Long COVID” and its association with on-going physical and respiratory symptoms in all adults discharged from hospital.. Eur. Respir. J..

[148] Sadeghizadeh-Yazdi J., Miri T., Mozaffari Nejad A.S. (2024). COVID-19 pandemic and food safety concerns: The impact of extra-heating on chemical toxins.. J. Food Qual. Hazards Control.

[149] Shahgolzari M., Yavari A., Arjeini Y., Miri S.M., Darabi A., Mozaffari Nejad A.S., Keshavarz M. (2021). Immunopathology and immunopathogenesis of COVID-19, what we know and what we should learn.. Gene Rep..

